# Seasonal characteristics and trend analysis of vector mosquitoes in Shandong province from 2018 to 2024

**DOI:** 10.3389/fcimb.2026.1753604

**Published:** 2026-04-08

**Authors:** Qintong Sun, Hao Yin, Xinyue Cao, Tong Cai, Yan Liu, Yingnan Han, Yingchun Yang, Chenxin Han, Hongli Zhao, Yumin Liang, Wenkui Sun, Hongmei Liu, Xuejun Wang

**Affiliations:** 1 Shandong Center for Disease Control and Prevention, Jinan, China; 2 Shandong Province Key Laboratory of Intelligent Monitoring, Early Warning, and Prevention of Infectious Diseases, Jinan, China; 3 Shandong Institute of Parasitic Diseases, Shandong First Medical University and Shandong Academy of Medical Sciences, Jining, China; 4 School of Public Health, Shandong First Medical University and Shandong Academy of Medical Sciences, Jinan, China; 5 Digestive Disease Hospital of Shandong First Medical University, Shandong Institute of Parasitic Diseases, Shandong First Medical University and Shandong Academy of Medical Sciences, Jining, China; 6 Jining Center for Disease Control and Prevention, Jining, China

**Keywords:** circular statistics, concentration method, joinpoint regression model, mosquito, seasonality

## Abstract

**Introduction:**

Mosquitoes are major vectors of various human pathogenic viruses, posing a significant and evolving public health threat. This is particularly true for China’s Shandong Province, which documented its northernmost local dengue fever outbreak in 2017, highlighting its vulnerability to sustained mosquito-borne disease transmission. In the context of the lack of universal vaccines, enhancing surveillance to inform precision control is critical. This study aims to characterize the population dynamics, seasonal patterns, and long-term trends of vector mosquitoes in Shandong Province from 2018 to 2024, thereby generating actionable evidence for targeted prevention strategies.

**Methods:**

Mosquito surveillance data collected across Shandong Province from 2018 to 2024 were systematically analyzed. The composition of mosquito vector assemblages was quantified and applied an integrated analytical framework: Degree of seasonal aggregation of mosquito populations were quantified using the concentration method, and peak timing, along with peak activity periods, was determined through circular statistical analysis. And the Joinpoint regression model was used to evaluate long-term trends in abundance and identify significant changes over the study period.

**Results:**

During the entire research period, *Culex pipiens pallens* was the predominant species in the monitoring work conducted in Shandong Province. From 2018 to 2024, the overall concentration degree of mosquito density in Shandong Province was M = 0.737, with γ = 0.722 (p < 0.05). The population peak occurred on July 30, and the peak activity period extended from June 13 to September 15. Significant regional variation was observed. The peak day occurred earliest in Linyi City, while Weihai recorded the latest peak day. From a long-term perspective, the province’s mosquito density exhibited a non-significant downward trend. Significant divergence in trends was observed between cities: Jinan, Yantai and Liaocheng exhibited marked decreases, whereas Weihai, Rizhao and Linyi showed significant increases.

**Conclusions:**

Vector mosquito populations in Shandong Province show distinct seasonal, with peak activity concentrated from mid-June to mid-September. *Culex pipiens pallens* was the dominant species. And regarding long-term trends, variations exist in mosquito density across cities within Shandong. These findings highlight the need for region-specific control measures and sustained surveillance to support precise prevention of mosquito-borne diseases.

## 1 Introduction

Mosquitoes serve as major vectors for numerous human pathogenic viruses (Onyango et al., 2020). Most medically important mosquito-borne pathogens belong to the families Flaviviridae, Togaviridae, and Bunyaviridae, as well as the parasite Plasmodium. They are responsible for mosquito-borne diseases such as dengue fever, malaria, and epidemic encephalitis B (Gaunt et al., 2001; Cheng et al., 2016; Huang et al., 2019). Dengue fever, transmitted by Aedes aegypti and Aedes albopictus, remains the most prevalent mosquito-borne infection globally (Bhatt et al., 2013). Additionally, Culex species, which are widely distributed across China, can transmit historically significant diseases such as bancroftian filariasis and Japanese encephalitis (Gao et al., 1999; Huang et al., 2006; Harshani and Abeynayake, 2025).

With rapid economic development in China, challenges such as urban population expansion, suboptimal housing conditions, and inadequately coordinated waste management systems have become increasingly evident. These factors create environments that favor mosquito breeding (Tian et al., 2016). In 2017, Shandong Province experienced a dengue fever outbreak initiated by an imported case and sustained through local transmission, representing the northernmost documented occurrence of locally acquired dengue at that time (Huang et al., 2023). Climate warming is reshaping the seasonal activity and spatial distribution of mosquito vectors, exerting significant impacts on public health and overall community wellbeing in both rural and urban settings worldwide (Lamy et al., 2023). Given the absence of vaccines or effective therapies for most mosquito-borne viruses, surveillance and control of vector populations remain essential public health strategies for limiting transmission (Wu et al., 2019). Such surveillance provides critical data on local vector abundance and supports the assessment of population-level risk (Wijegunawardana et al., 2019).

Advanced analytical methods are essential to extract actionable insights from surveillance data. Recently, the concentration method, circular statistics, and the Joinpoint regression model have been increasingly used to characterize the epidemic dynamics and temporal patterns of vector-borne organisms and their associated infectious diseases. Analysis of adult mosquito seasonal trends involves plotting curves based on monitoring counts or density for qualitative description. This method is notably simple to operate and provides intuitive, effective results. However, it is highly subjective and has significant limitations: it cannot determine the peak day or period of seasonal fluctuations, nor can it reliably assess statistical significance. The concentration method is applied to assess whether a given disease shows distinct seasonality. Circular statistics are then used to accurately determine the annual onset and peak periods, guiding targeted short-term prevention and control measures. Finally, the Joinpoint regression model analyzes multi-year incidence data to identify shifts in long-term trends and evaluate the effectiveness of intervention strategies. The integrated application of these three approaches is critical for optimizing the prevention and control of vector-borne infectious diseases (Zeng, 2019; Han et al., 2021; Li et al., 2022; Wang et al., 2025). Currently, the application of this methodology in Shandong Province remains limited, which, to some extent, restricts long-term and comprehensive analyses of mosquito distribution patterns in the region.

To address this gap, the study aimed to characterize the seasonal patterns and temporal trends of vector mosquito populations in Shandong Province (2018–2024) using concentration analysis, circular statistics, and Joinpoint regression, to improve the identification of peak activity periods and strengthen risk assessment, surveillance, and targeted vector control strategies.

## 2 Materials and methods

### 2.1 Mosquito detection

Base on the Shandong Province Vector Surveillance Implementation Plan, adult mosquitoes were monitored from March to November across 16 prefecture-level cities in Shandong Province, including Jinan, Qingdao, Zibo, Zaozhuang, Dongying, Yantai, Weifang, Jining, Tai’an, Weihai, Rizhao, Linyi, Dezhou, Binzhou, Liaocheng, and Heze. Surveillance was conducted in seven types of habitats: residential areas, parks, hospitals, farmlands, cowsheds, pigsties, and breeding farms. When selecting sampling points, a combined strategy of stratified sampling and random sampling is adopted to determine the specific sampling points, in order to ensure the representativeness and feasibility of the samples in terms of geographical distribution. The same set of fixed sites within each city and habitat were monitored annually throughout the study period by dedicated staff from local county-level CDCs, ensuring the consistency and comparability of the longitudinal data.​ Monthly mosquito surveillance data from 2018 to 2024 were obtained from the national key vector surveillance system. The dataset included information on surveillance date, habitat type, the number of captured female mosquitoes, mosquito density, and species composition.

### 2.2 Research methods

#### 2.2.1 Mosquito trap method

Adult mosquitoes were monitored according to the standardized procedures described in the national guideline Surveillance Methods for Vector Density—Mosquitoes (GB/T 23797-2020). The photocatalytic mosquito and fly trap, commercially known as “Gongfu Xiaoshuai” (manufactured by Wuhan Jixing Environmental Protection Technology Co., Ltd., package dimensions: 22 * 22 * 36 cm), was employed for sampling. The device does not utilize carbon dioxide as an attractant. It was deployed outdoors, operational daily from approximately 17:00 to 08:00 the following morning, and was installed at a height of 1.5 meters. Captured specimens were then euthanized, and species identification and sex determination were conducted using established morphological keys. The mosquito density was calculated by the formula:


$$\text{Mosquito density }(\text{females}/\text{trap night})=\frac{\text{number of females}}{\text{Number of lights}\times \text{Number of nights}}$$

#### 2.2.2 Concentration method

The concentration method is a statistical approach used to quantify the seasonality of mosquito populations by integrating monthly density data over a complete annual cycle (Zhao et al., 2020a; Duan et al., 2023; Wilder-Smith, 2024). The calculation was performed using the following formula:


$$\text{M }=\sqrt{{\text{R}}_{\text{x}}^{2}+{\text{R}}_{\text{y}}^{2}}\;$$

$${\text{R}}_{\text{x}}=\frac{\left({\text{r}}_{2}+{\text{r}}_{6}-{\text{r}}_{8}-{\text{r}}_{12}\right)}{2}+\sqrt{3}\frac{\left({\text{r}}_{3}+{\text{r}}_{5}-{\text{r}}_{9}-{\text{r}}_{11}\right)}{2}+({\text{r}}_{4}-{\text{r}}_{10})$$

$${\text{R}}_{\text{y}}=\frac{\left({\text{r}}_{3}-{\text{r}}_{5}-{\text{r}}_{9}+{\text{r}}_{11}\right)}{2}+\sqrt{3}\frac{\left({\text{r}}_{2}+{\text{r}}_{12}-{\text{r}}_{6}-{\text{r}}_{8}\right)}{2}+({\text{r}}_{1}-{\text{r}}_{7})$$

In the formula, M and R represent concentration and dispersion, respectively, while r_i_ (i = 1, 2,…,12) denotes the proportion of mosquitoes captured in each month relative to the total annual captures. Seasonal classification is based on the value of M:

M ≥ 0.9: strict seasonality
0.7 ≤ M < 0.9: strong seasonality
0.5 ≤ M < 0.7: moderate seasonality
0.3 ≤ M < 0.5: weak seasonality
M < 0.3: minimal or no seasonality

#### 2.2.3 Circular statistics

Circular statistics is a widely used method for analyzing seasonal patterns (Zhao et al., 2020a; Duan et al., 2023). This approach converts periodic (circular) data into linear form using trigonometric transformations and employs γ to represent the concentration of the circular distribution. The calculation is performed using the following formula:


$$\text{x}=\frac{\sum \text{f}\cos\text{α}}{\text{n}}$$

$$\text{y}=\frac{\sum \text{f}\sin\text{α}}{\text{n}}$$

$$\text{γ}=\sqrt{{\text{x}}^{2}+{\text{y}}^{2}}\;$$

$$\cos\bar{\text{α}}=\frac{\text{y}}{\gamma }$$

$$\sin\bar{\text{α}}=\frac{\text{x}}{\gamma }$$

$$\text{Z}={\text{nγ}}^{2}$$

$$\text{S}=\frac{180{}^{\circ}}{\text{π}\sqrt{-2\ln\text{γ}}}$$

$$\bar{\text{α}}=\{\begin{array}{ll} \text{arctg}(\frac{\text{y}}{\text{x}}) & x>0,\text{y}>0\\ 180{}^{\circ}+\text{arctg}(\frac{\text{y}}{\text{x}}) & x<0\\ 360{}^{\circ}+\text{arctg}(\frac{\text{y}}{\text{x}}) & x>0,\text{y}<0 \end{array}$$

$$\text{peak day}=\bar{\text{α}}*(365/360)$$

$$\text{peak period}=\bar{\text{α}}\pm \text{S}*(365/360)$$

In this formula, f represents the monthly mosquito density, α is the angle corresponding to the median value of the dataset (median angle), n is the total mosquito density, γ denotes the central tendency of the circular distribution, S is the circular standard deviation or mean angular deviation, and represents the mean angle of the sample. The Rayleigh test was applied to assess the presence of a significant mean angle. For sufficiently large sample sizes (e.g., > 100), the 95% confidence interval (CI) of the mean angle (± S) was calculated to estimate the peak period of mosquito activity. A test statistic Z > Z0.05 indicates statistical significance.

#### 2.2.4 Joinpoint regression model

Temporal trends in annual mosquito density from 2018 to 2024 were analyzed using a Joinpoint regression model implemented in the Joinpoint Regression Program (v.5.3.0.0). The model was used to estimate the annual percentage change (APC) and the average annual percent change (AAPC), along with their 95% CIs. An APC or AAPC > 0 indicates an increasing trend, a value < 0 indicates a decreasing trend, and a value not significantly different from zero indicates a stable trend. Since the Joinpoint model requires non-zero observations, zero values in the dataset were replaced with 0.001.

### 2.3 Statistical analysis

Data collation, analysis, and visualization were performed using WPS 2022 software. Circular statistics were computed manually based on standard formulas within the WPS environment. Differences in the composition ratios of mosquito species across various habitat types were assessed using the χ² test. Temporal distribution patterns were evaluated using the concentration method, circular statistics, and Joinpoint regression modeling. The monitoring data for each group were subjected to the von Mises distribution goodness-of-fit test. If the test results for all groups showed p > 0.05, the data were considered to meet the von Mises distribution assumption, and the Watson-Williams test was used for intergroup comparisons. If any group had p ≤ 0.05, the assumption of conforming to the von Mises distribution was rejected, and the non-parametric Mardia-Watson-Wheeler test was applied. The Mann-Kendall trend test was used to analyze the trend in mosquito density. The significance level was set at α = 0.05.

## 3 Results

### 3.1 Mosquito density and species composition in various habitats of Shandong province

**Figure 1:**
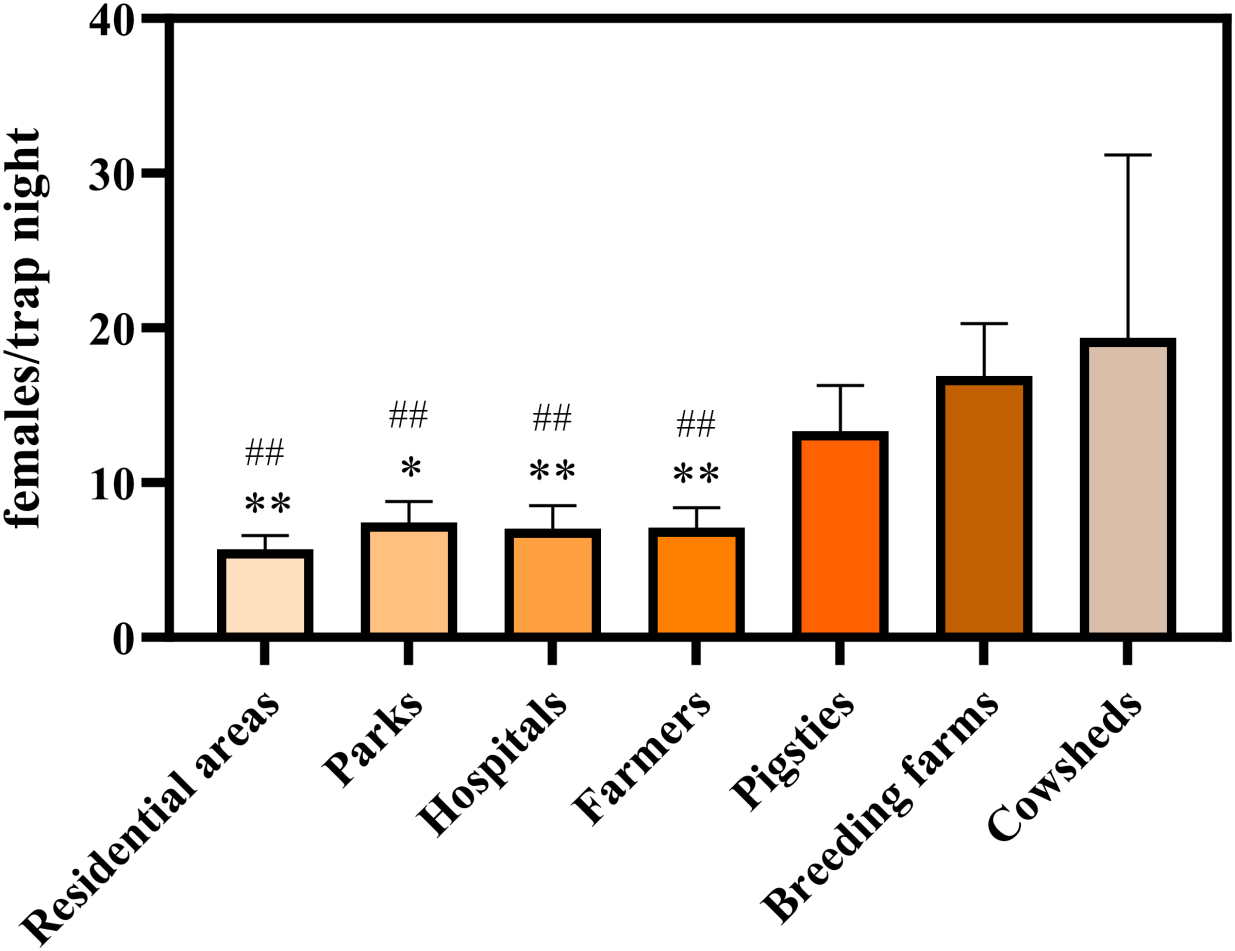
Differential mosquito density across habitat types ##p < 0.01, compared to the cowsheds. *p < 0.05, **p < 0.01, compared to the breeding farms.

**Figure 2:**
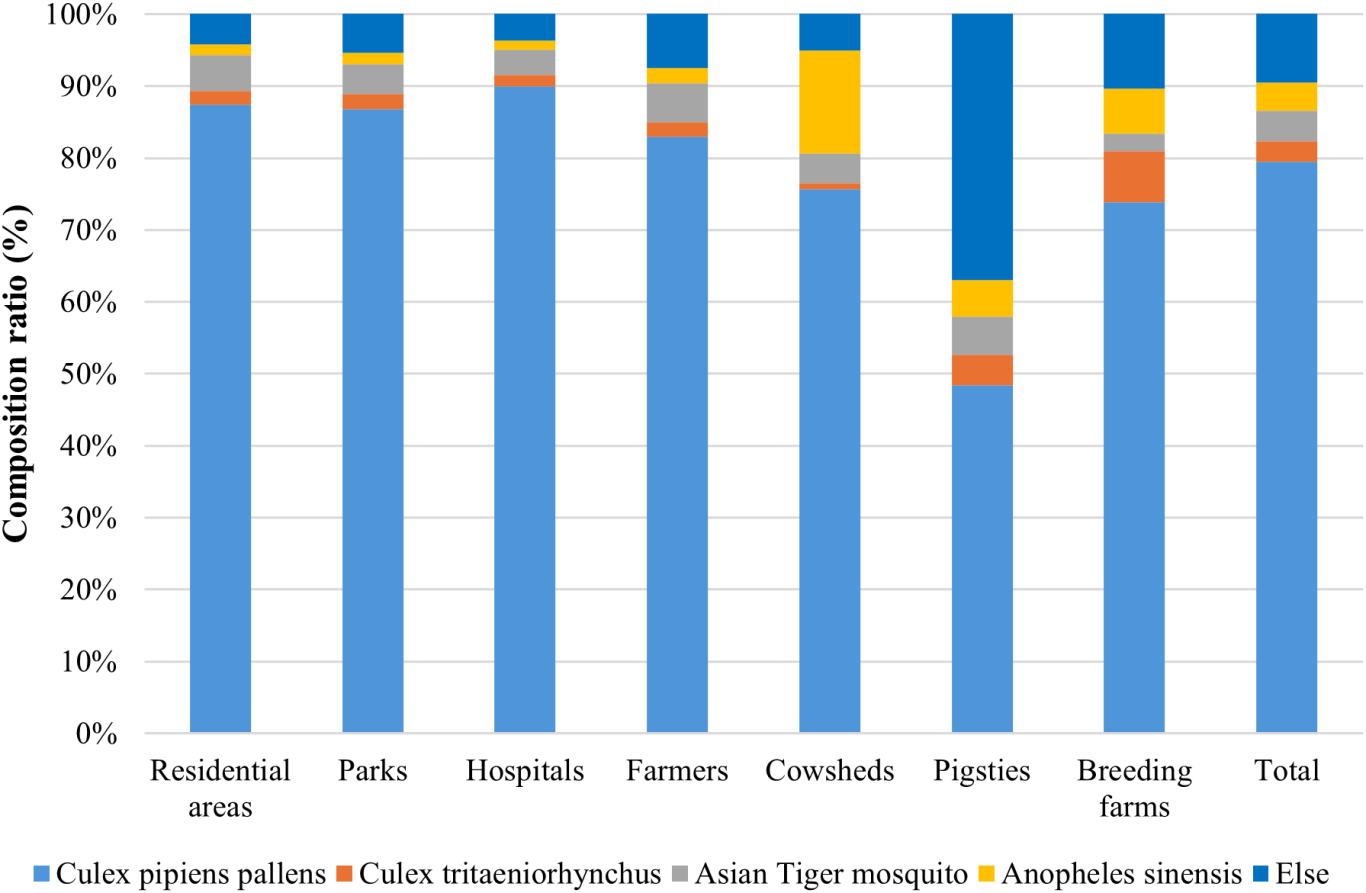
Composition and proportional distribution of mosquito species.

From 2018 to 2024, a total of 273,041 female adult mosquitoes were captured across monitoring sites in Shandong Province, representing eight species within four genera. Mosquito densities were highest in cattle sheds, livestock farms, and pigsties, with mean densities of 18.14, 16.62, and 12.93 females per trap night (**Figure 1**). The species composition varied significantly across habitat types (χ² = 47,757.65, df = 24, p < 0.001; Cramer’s V = 0.209). Subsequent *post-hoc* analysis, based on adjusted residuals (|Adjusted Residual| > 2.0), revealed specific preferences: *Culex pipiens pallens* was significantly overrepresented in residential areas, parks, hospitals, and rural households but underrepresented in pigsties (**Supplementary Table S1**). Overall, *Culex pipiens pallens* was the predominant species overall (79.44%), though its proportion varied widely by habitat, from 48.38% to 89.98%(**Figure 2**, **Supplementary Table 2**).

### 3.2 Concentration analysis of mosquitoes

**Figure 3:**
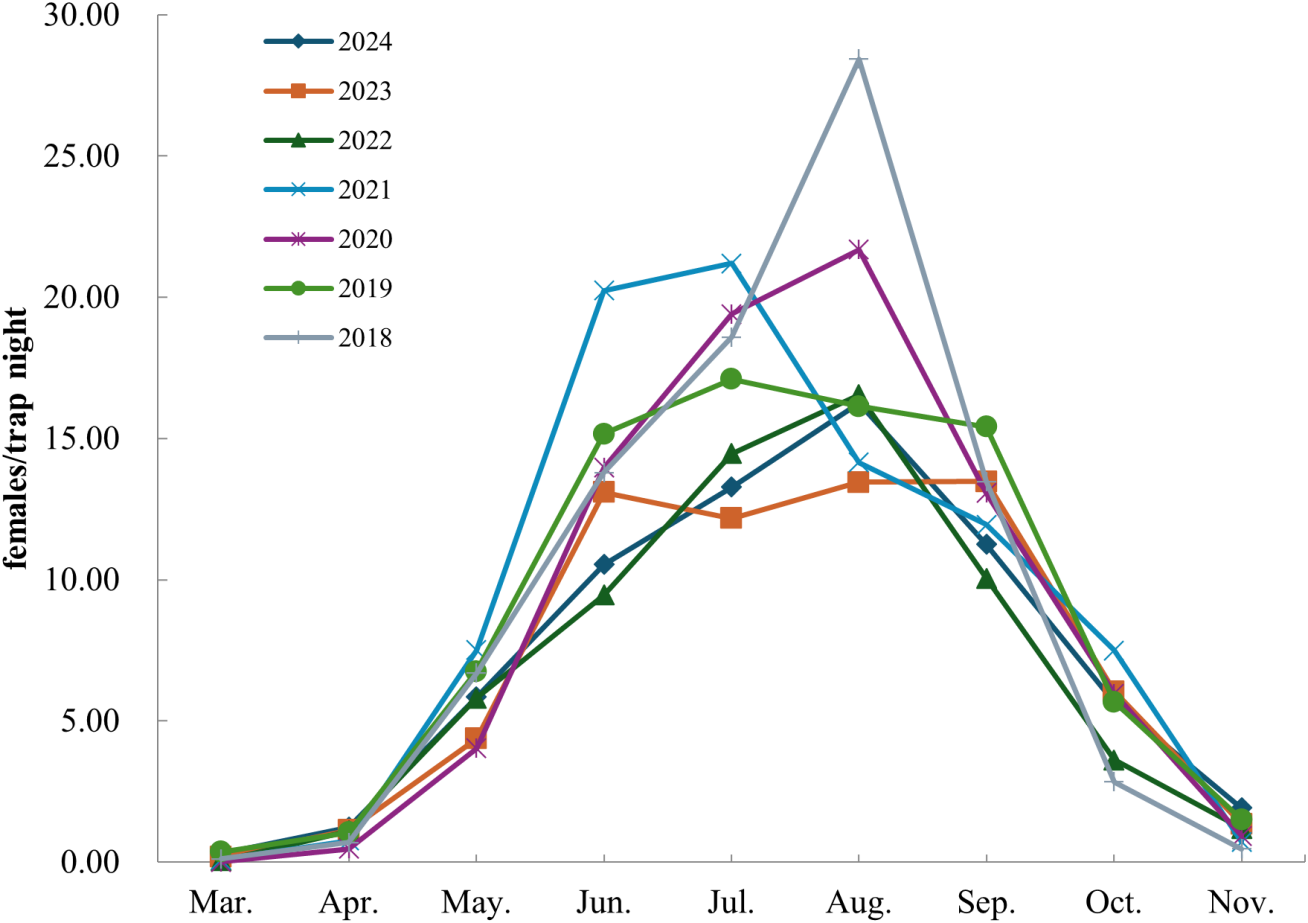
The trend of increase and decrease in adult mosquito density in Shandong Province from 2018 to 2024.

Mosquito populations in Shandong Province have recently displayed a unimodal seasonal pattern. Mosquitoes begin to appear in March, with numbers gradually increasing after May. A sharp rise in population occurs by June, and adult mosquito densities peak between June and September, with July and August showing the highest densities. From October onward, mosquito abundance declines steadily, with populations largely disappearing by mid-to-late November (**Figure 3**).

Using the concentration formula, R_x_ was calculated as -0.174, R_y_ as -0.716, and the overall mosquito monitoring density concentration M as 0.737. Across the period from 2018 to 2024, annual M values ranged from 0.692 to 0.786, indicating a significant seasonal pattern in mosquito populations (**Table 1**).

**Table 1:** Mosquito monitoring density and concentration in Shandong province from 2018 to 2024.

Month	2018	2019	2020	2021	2022	2023	2024	Total
Mar.	0.11	0.37	0.01	0.09	0.05	0.05	0.31	0.99
Apr.	0.69	1.04	0.47	0.76	1.1	0.94	1.23	6.23
May.	6.56	6.75	4.02	7.5	5.8	5.4	5.85	41.88
Jun.	13.52	15.16	13.96	20.66	9.45	9.05	10.54	92.34
Jul.	18.25	17.1	19.4	21.45	14.46	14.15	13.28	118.09
Aug.	28.43	16.13	21.68	15.42	16.54	16.16	16.25	130.61
Sep.	13.47	15.41	13.06	12.58	10.04	9.53	11.24	85.33
Oct.	2.84	5.68	5.96	6.57	3.62	3.26	5.66	33.59
Nov.	0.46	1.5	0.93	0.87	1.19	1.1	1.92	7.97
Rx	-0.188	-0.172	-0.226	-0.096	-0.172	-0.174	-0.201	-0.174
Ry	-0.763	-0.687	-0.734	-0.725	-0.711	-0.719	-0.663	-0.716
M	0.786	0.708	0.768	0.731	0.732	0.74	0.692	0.737

### 3.3 The peak season for mosquitoes

#### 3.3.1 Analysis of peak days and peak periods of mosquito density in Shandong province

Circular distribution analysis of monthly mosquito density in Shandong Province from 2018 to 2024 is summarized in **Table 2**. Based on the circular distribution analysis, the aggregated data over the seven years yielded X = -0.638 and Y = -0.340, with a g-value of 0.722. The mean angle (α) was located in the third quadrant. The Rayleigh test confirmed statistically significant clustering (Z = 38.13, P < 0.001), indicating the presence of a mean angle. The von Mises distribution goodness-of-fit test resulted in P < 0.05, leading to the application of the Mardia-Watson-Wheeler non-parametric test. The results showed W = 5.329, P = 0.946, suggesting no statistically significant differences in the mean angles of mosquito density across the years. With α = 208.027° and S = 46.219°, and according to the principles of circular statistics, the overall peak day of mosquito density in Shandong Province occurred on 30 July, and the peak period spanned from 13 June to 15 September, lasting approximately three months (**Table 3**).

**Table 2:** Calculation results of circular distribution of mosquito monitoring density in Shandong province from 2018 to 2024.

Month	Monthly median	Average angle (°)	sinα	cosα	Monthly monitoring density	fsinα	fcosα
Jan.	15.5	15.29	0.264	0.965	0.001	0.000	0.001
Feb.	45	44.38	0.699	0.715	0.001	0.001	0.001
Mar.	74.5	73.48	0.959	0.284	0.986	0.945	0.280
Apr.	105	103.56	0.972	-0.234	6.234	6.061	-1.462
May.	135.5	133.64	0.724	-0.690	41.878	30.305	-28.903
Jun.	166	163.73	0.280	-0.960	92.340	25.876	-88.640
Jul.	196.5	193.81	-0.239	-0.971	118.086	-28.184	-114.674
Aug.	227.5	224.38	-0.699	-0.715	130.612	-91.358	-93.345
Sep.	258	254.47	-0.963	-0.268	85.334	-82.217	-22.854
Oct.	288.5	284.55	-0.968	0.251	33.587	-32.510	8.437
Nov.	319	314.63	-0.712	0.703	7.972	-5.673	5.601
Dec.	349.5	344.71	-0.264	0.965	0.001	0.000	0.001
Total						-176.754	-335.557

**Table 3:** Peak days and periods of mosquito monitoring density in Shandong province from 2018 to 2024.

Year	$\bar{\text{α}}$°	S°	Peak days	Peak periods	γ	*Z*
2018	208.010	40.144	Jul. 30th	Jun. 19th - Sep. 9th	0.782	51.616
2019	208.145	48.001	Jul. 31st	Jun. 12th - Sep. 18th	0.704	39.224
2020	211.240	41.982	Aug. 3rd	Jun. 21st - Sep.15th	0.765	46.470
2021	201.558	45.705	Jul. 24th	Jun. 7th - Sep. 9th	0.727	45.461
2022	207.718	45.642	Jul. 30th	Jun. 13th - Sep. 15th	0.728	33.003
2023	207.685	44.858	Jul. 30th	Jun. 14th - Sep. 14th	0.736	32.310
2024	211.036	49.496	Aug. 2nd	Jun. 12th - Sep. 22nd	0.689	31.425
Total	208.027	46.219	Jul. 30th	Jun. 13th - Sep. 15th	0.722	38.131

#### 3.3.2 Analysis of peak days and periods of mosquito density in various regions of Shandong province

Analysis of mosquito density data from 16 prefecture-level cities between 2018 and 2024 revealed regional differences in peak activity. The mosquito surveillance data underwent a von Mises distribution goodness-of-fit test, resulting in P<0.05. Therefore, the Mardia-Watson-Wheeler non-parametric test was applied. The results showed W = 31.586, P = 0.011, indicating a statistically significant difference in the mean angles of mosquito density among the 16 prefecture-level cities. The peak days of mosquito density varied across different localities. According to the principles of circular statistics, the earliest peak of mosquito activity in Shandong Province occurred in Linyi City in the southern Shandong region, with the peak day on July 16th and the peak period from June 1st to August 30th. This was followed by inland areas such as Liaocheng, Tai’an, Weifang, and Heze. The peak days in coastal areas were slightly later than those inland, with the latest occurring in Weihai City, where the peak day was August 22nd and the peak period spanned from July 11th to October 3rd (**Table 4**).

**Table 4:** Peak days and periods of mosquito density in each city.

City	$\bar{\text{α}}$°	S°	Peak days	Peak periods	γ	Z
Jinan	208.350	36.562	Jul. 31st	Jun. 23rd – Sep. 7th	0.816	35.468
Qingdao	213.059	40.658	Aug. 5th	Jun. 24th – Sep. 16th	0.777	34.420
Zibo	209.307	48.905	Aug. 1st	Jun. 12th – Sep. 20th	0.695	26.867
Zaozhuang	214.459	47.735	Aug. 6th	Jun. 18th – Sep. 24th	0.707	71.955
Dongying	208.951	40.937	Jul. 31st	Jun. 19th – Sep. 11th	0.775	20.323
Yantai	215.023	46.775	Aug. 7th	Jun. 20th – Sep. 24th	0.717	17.963
Weifang	205.191	36.645	Jul. 27th	Jun. 19th – Sep. 3rd	0.815	18.739
Jining	213.646	49.420	Aug. 5th	Jun. 15th – Sep. 25th	0.689	23.875
Taian	202.951	52.882	Jul. 25th	Jun. 1st – Sep. 17th	0.653	18.927
Weihai	231.579	41.015	Aug. 22nd	Jul. 11th – Oct. 3rd	0.774	53.343
Rizhao	210.292	42.358	Aug. 1st	Jun. 19th – Sep. 13th	0.761	26.000
Linyi	193.465	44.374	Jul. 16th	Jun. 1st – Aug. 30th	0.741	92.218
Dezhou	213.162	34.466	Aug. 5th	Jul. 1st – Sep. 9th	0.834	28.405
Liaocheng	199.314	43.579	Jul. 22nd	Jun. 8th – Sep. 4th	0.749	24.098
Binzhou	211.82	42.301	Aug. 2nd	Jun. 20th – Sep. 14th	0.761	58.449
Heze	208.273	55.958	Jul. 30th	Jun. 3rd – Sep. 25th	0.621	32.346
Total	208.027	46.219	Jul. 30th	Jun. 13th – Sep. 15th	0.722	38. 131

### 3.4 Annual variation trend of mosquito monitoring density

**Figure 4:**
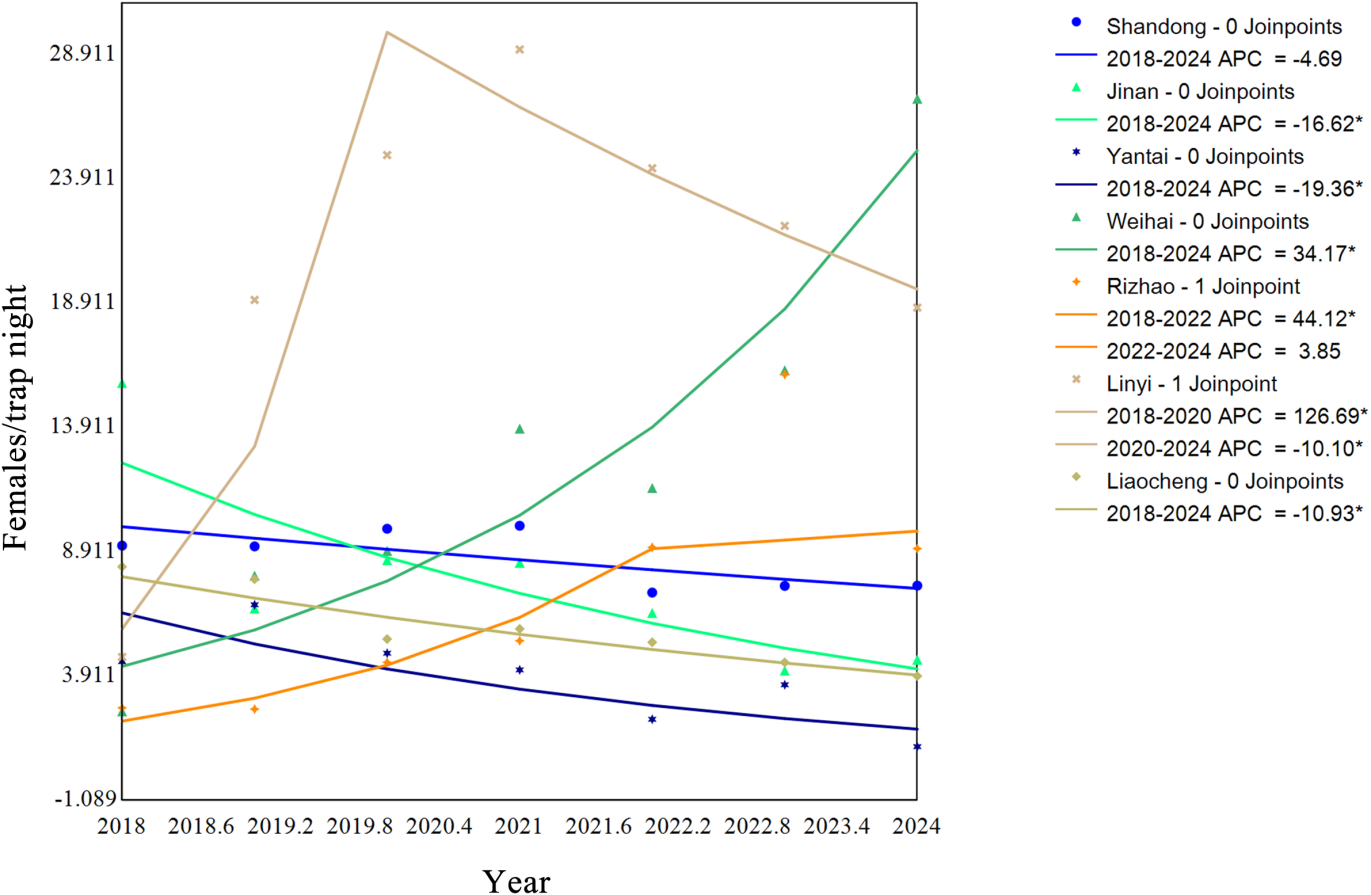
Annual variation trend of mosquito monitoring density in Shandong Province and some cities from 2018 to 2024.

Joinpint regression analysis was conducted on mosquito densities in Shandong Province from 2018 to 2024 (**Figure 4**; **Table 5**). At the provincial level, the average annual percentage change (AAPC) in mosquito density was -4.691% (95% CI: -9.29% to 0.14%, P = 0.055), indicating that the overall trend was not statistically significant. However, significant differences were observed in the long-term trends of individual cities. The mosquito densities in Weihai, Rizhao, and Linyi cities showed significant increasing trends, with AAPCs of 34.167%, 20.566%, and 22.366%, respectively. In contrast, significant decreasing trends were found in Jinan, Yantai, and Liaocheng cities, with AAPCs of -16.623%, -19.364%, and -10.930%, respectively. The mosquito densities in the remaining 11 cities did not exhibit a significant monotonic increasing or decreasing trend during the study period. Further analysis of cities with significant increasing trends revealed that their internal change patterns were not purely linear (**Table 6**). For example, in Linyi City, mosquito density increased sharply from 2018 to 2020 (APC = 126.69%) but subsequently shifted to a significant decline from 2020 to 2024 (APC=-10.10%). In Rizhao City, mosquito density rose rapidly from 2018 to 2022 (APC = 36.32%) before stabilizing thereafter.

**Table 5:** Annual average trend in mosquito density in Shandong province, 2018–2024.

City	AAPC (95%CI) %	Z	P
Jinan	-16.623 (-25.67, -6.48)	-4.070	0.010
Qingdao	-8.832 (-19.92, 3.80)	-1.832	0.126
Zibo	1.410 (-14.10, 19.73)	0.217	0.836
Zaozhuang	-2.585 (-9.17, 4.47)	-0.734	0.463
Dongying	0.216 (-7.82, 8.95)	0.051	0.960
Ynatai	-19.364 (-33.23, -2.61)	-2.931	0.033
Weifang	6.328 (-23.55, 47.89)	0.478	0.653
Jining	-8.569 (-21.72, 6.80)	-1.482	0.198
Taian	-8.404 (-31.83, 23.07)	-0.764	0.479
Weihai	34.167 (15.38, 56.02)	5.007	0.004
Rizhao	20.566 (5.45, 37.85)	2.736	0.006
Linyi	22.366 (19.68, 25.12)	17.794	< 0.001
Dezhou	-6.361 (-42.25, 51.83)	-0.267	0.790
Liaocheng	-10.930 (-14.93, -6.74)	-6.471	0.001
Binzhou	-8.800 (-18.18, 1.65)	-2.182	0.081
Hze	-3.371 (-40.01, 55.64)	-0.141	0.888
Total	-4.691 (-9.29, 0.14)	-2.497	0.055

**Table 6:** Annual trends in mosquito density in Rizhao and Linyi cities.

City	Time period	APC (95%CI) %	t	P
Rizhao	2018-2022	36.32 (4.85, 77.23)	5.079	0.037
	2022-2024	-5.69 (-53.59, 91.67)	-0.355	0.756
Linyi	2018-2020	126.69 (101.63, 154.85)	30.064	0.001
	2020-2024	-10.10 (-13.96, -6.06)	-10.422	0.009

## 4 Discussion

Mosquitoes transmit a range of pathogens through hematophagy, resulting in hundreds of millions of infections annually and constituting a major threat to global public health (Ali, 2023). The World Health Organization estimates that mosquito-borne diseases account for more than 700,000 deaths each year (WHO, 2023). Ongoing climate warming influences all developmental stages of mosquitoes, sustaining the risk of both imported and locally acquired outbreaks of dengue, malaria, and other vector-borne infections in China (Li et al., 2013; Oberlin and Wylie, 2023). Continuous, standardized surveillance is therefore essential. In this study, mosquito monitoring data from 2018 to 2024 were compiled across multiple habitat types in Shandong Province, including urban residential zones, parks, sanitation sites, rural households, and livestock structures. These data support the analysis of mosquito population dynamics and provide an evidence base for designing targeted vector control strategies.

In this study, *Culex pipiens pallens* was the dominant mosquito species in Shandong Province, representing 79.44% of all captured specimens. Mosquito densities were highest in rural livestock-related environments, including cattle sheds, pigsties, and breeding facilities. This pattern is consistent with the 2019 national vector surveillance report; however, the proportion of the dominant species in Shandong Province surpasses the national mean (Zhao et al., 2020b). Statistical analysis confirmed significant variation in species composition across habitats (χ² = 47,757.65, df = 24, p < 0.001; Cramer’s V = 0.209), with *Culex pipiens pallens* showing a significant preference for residential areas, parks, hospitals, and rural households (|adjusted residual| > 2.0) but underrepresentation in pigsties (**Supplementary Table S1**).​​ *Culex pipiens pallens*, the dominant mosquito species in northern China, typically breeds in polluted water bodies near residential areas, including ditches and septic tanks. Suboptimal sanitation in livestock environments, insufficient wastewater management, and the availability of vertebrate hosts facilitate its abundance (Victor et al., 2017; Waite et al., 2017; Zhao et al., 2020b). Post-elimination surveillance in Shandong Province by Li Yuejin et al. similarly identified *Culex* sp*ecies* as predominant, accounting for 92.26% of captured specimens, a pattern that may reflect habitat selection bias inherent in the monitoring framework (Li et al., 2024b).

High mosquito densities in rural aquaculture and similar habitats represent a priority target for vector control and are consistent with surveillance findings reported in multiple provinces (Ding et al., 2021; Yang et al., 2021). Compared with pale-colored *Culex* species, the proportion of other taxa such as *Aedes albopictus* is relatively low, potentially reflecting lower trap sensitivity for this species (Yang et al., 2015; Xiong et al., 2019). As one of the most widespread mosquito species in China, *Aedes albopictus* can transmit several major arboviruses, including chikungunya fever, dengue, and Zika viruses, underscoring the need for comprehensive monitoring and control efforts (Gratz, 2004; Liu et al., 2019).

Circular statistical methods allow quantitative characterization of periodicity and are widely used to assess seasonal patterns in disease incidence, mortality, and vector density, facilitating analysis of temporal clustering and seasonal dynamics (Wang and Li, 2006; Li et al., 2024a). In this study, circular distribution analysis identified 30 July as the peak activity date for mosquitoes in Shandong Province, with the peak period extending from 13 June to 15 September.

Time-trend analysis using the Joinpoint regression model enabled detailed evaluation of temporal changes across the study period by segmenting observations, fitting segment-specific trends, and identifying trend inflection points (Li and Du, 2020). The seasonal patterns observed in this study are consistent with previous investigations. Yuan et al. reported peak mosquito densities in July in wildlife and central urban parks in Shanghai, with suburban forest parks peaking in August, and identified *Culex pipiens pallens* and *Aedes albopictus* as the predominant species (Yuan et al., 2025). Similarly, Lv et al. found that *Anopheles sinensis* density in Shandong Province peaked in July and August (Lv et al., 2024). Han et al. reported a peak on 2 August in Hebei Province, with the peak period spanning 27 June to 5 September (Han et al., 2021). These comparisons suggest that variation in peak mosquito activity across regions is influenced by latitudinal and ecological differences. Circular statistical analysis in this study provides more accurate estimates of peak timing and seasonal duration compared with traditional linear approaches, supporting targeted vector surveillance and control strategies.

Shandong Province, situated in central and eastern coastal China, falls within the warm-temperate monsoon climate zone. Coastal areas experience a later seasonal onset compared with inland regions at similar latitudes due to maritime influence. Temperature gradients are more significant along the east–west axis than the north–south axis, resulting in distinct climatic differences between coastal and inland areas (Zhu et al., 2021). Annual maximum temperatures are higher in western Shandong and lower in the eastern coastal regions, with the frequency of hot days, warm nights, and heatwave events also following an east–west gradient, being greater in the west (Huan et al., 2025). Correspondingly, the peak of mosquito activity in the Jiaodong Peninsula typically occurs later than in western inland areas and has a slightly shorter duration compared with inland cities. The delay in peak mosquito activity is most significant in Weihai, a coastal city in eastern Shandong surrounded by sea on three sides, where the peak occurs on 22 August, approximately one month later than in inland cities such as Jinan and Liaocheng. This climatic variation exhibits a high degree of spatial correspondence with our findings, and we therefore propose it as a potential explanation for regional disparities in mosquito distribution. As this study did not undertake specific correlation analyses, we discuss this possibility here without establishing it as evidence for a quantitative causal relationship. Although meridional temperature variation across Shandong Province is minimal, surveillance data indicate that peak activity begins earlier in southern Linyi City (16 July), roughly half a month before northwestern cities, including Dezhou, Binzhou, and Dongying. The earlier peak observed in southern cities spatially aligns with the known southeast-to-northwest precipitation gradient in Shandong (Ren et al., 2024). Although this study did not directly analyze rainfall data, the spatial consistency suggests that higher precipitation in the south may be a potential factor contributing to the earlier onset of mosquito activity. This hypothesis requires further validation through comprehensive meteorological data.

Mosquito surveillance across Shandong Province reveals heterogeneous annual trends in population density. Declining densities were observed in Jinan, Yantai, and Liaocheng, whereas Rizhao and Linyi showed an initial increase followed by a subsequent decline. In comparison, Weihai consistently displayed rising mosquito densities. These regional differences may reflect variations in vector control efforts. In urban areas, older residential districts contain numerous water-holding structures that support mosquito breeding, requiring intensified control measures (Wu and Chu, 2022). Rural areas, however, have benefited from recent environmental improvements and effective elimination of breeding sites, contributing to overall reductions in mosquito density (Yang et al., 2021).

This study has several limitations. Firstly, the utilization of light traps might have led to an under-sampling of phototaxis-weak species, such as Aedes albopictus. This potential bias could have influenced the reported species composition. Secondly, the analysis did not incorporate confounding variables, such as detailed local precipitation records, socioeconomic data, or specifics of vector control interventions. This omission constrains a comprehensive interpretation of the observed regional trends. It is important to note that the monitoring period from March to November may not fully capture potential shifts in seasonal activity, such as extended seasons or overwintering in microhabitats, driven by climate warming. Notwithstanding these limitations, the study establishes a valuable foundation for understanding mosquito population dynamics in Shandong Province.

## 5 Conclusions

This study characterized the seasonal dynamics of mosquito activity and examined annual trends in mosquito densities across Shandong Province, providing a scientific foundation for targeted vector control and the management of mosquito-borne diseases. In conclusion, mosquito populations in Shandong Province show distinct seasonal and regional patterns, with peak activity occurring from mid-June to mid-September. *Culex pipiens pallens* was the dominant species, particularly abundant in rural livestock-related habitats such as cattle sheds and pigsties. These findings align with national surveillance data and previous regional studies, although the proportion of *Culex pipiens pallens* in Shandong exceeds the national mean. Seasonal and spatial variation appears to be influenced by climatic gradients, habitat characteristics, and differences in vector control efforts. Regarding long-term trends, variations exist in mosquito density across cities within Shandong Province. Firstly, the provincial average mosquito density remains stable. However, distinct trends emerge among individual cities: Weihai, Rizhao, and Linyi exhibit a marked upward trajectory in mosquito density, with Weihai experiencing a persistent increase in mosquito numbers. This indicates these areas have become priority zones requiring enhanced control measures. Conversely, mosquito densities have markedly decreased in Jinan, Yantai, and Liaocheng. The results underscore the need for region-specific control measures and sustained surveillance to enable effective prevention of mosquito-borne diseases, particularly in high-density rural and aquaculture environments.

## Data Availability

The original contributions presented in the study are included in the article/**Supplementary Material**. Further inquiries can be directed to the corresponding authors.
